# Treatment of malaria from monotherapy to artemisinin-based combination therapy by health professionals in rural health facilities in southern Cameroon

**DOI:** 10.1186/1475-2875-8-174

**Published:** 2009-07-29

**Authors:** Collins Sayang, Mathieu Gausseres, Nicole Vernazza-Licht, Denis Malvy, Daniel Bley, Pascal Millet

**Affiliations:** 1 Department of Tropical Medicine, Centre René Labusquière, University of Bordeaux2, 146 rue Léo Saignat 33076 Bordeaux Cedex France; 2 Department of Anthropology, DESMID – UMR 6012 Espace, 1, rue Parmentier – 13200 Arles, France

## Abstract

**Background:**

One year after the adoption of artesunate-amodiaquine (AS/AQ) as first-line therapy for the treatment of uncomplicated malaria, this study was designed to assess the treatment practices regarding anti-malarial drugs at health facilities in four rural areas in southern Cameroon.

**Methods:**

Between April and August 2005, information was collected by interviewing fifty-two health professionals from twelve rural health facilities, using a structured questionnaire.

**Results:**

In 2005, only three anti-malarial drugs were used in rural health facilities, including: amodiaquine, quinine and sulphadoxine-pyrimethamine. Only 2.0% of the health professionals prescribed the recommended AS/AQ combination. After reading the treatment guidelines, 75.0% were in favour of the treatment protocol with the following limitations: lack of paediatric formulations, high cost and large number of tablets per day. Up to 21.0% of professionals did not prescribe AS/AQ because of the level of adverse events attributed to the use of amodiaquine as monotherapy.

**Conclusion:**

The present study indicates that AS/AQ was not available in the public health facilities at the time of the study, and health practitioners were not informed about the new treatment guidelines. Results of qualitative analysis suggest that prescribers should be involved as soon as possible in projects related to the optimization of treatment guidelines and comply with new drugs. Adapted formulations should be made available at the international level and implemented locally before new drugs and treatments are proposed through a national control programme. This baseline information will be useful to monitor progresses in the implementation of artemisinin-based combination therapy in Cameroon.

## Background

Implementation of new drug policies is a complex issue, especially in poor rural areas where nurses are used to prescribe well-known drugs, readily available, inexpensive and easy to administer [1]. Few studies have investigated the adherence of medical practitioners to drug policy changes and new treatment protocols based on the use of artemisinin-based combination therapy (ACT). This work was carried out in two parts. In a previous study, the availability of anti-malarial drugs, treatment practices and acceptability of the new protocol by health professionals, have been assessed in urban health facilities in Yaoundé, capital city of Cameroon [2]. The present study analyses attitudes and practices of medical doctors and nurses regarding the treatment of malaria in rural health facilities in southern Cameroon. Specific objectives describe specific prescription patterns of anti-malarial drugs to adults, pregnant women and under five children, including attitudes and perception towards the new treatment guidelines based on ACT.

## Methods

### Setting

The survey was conducted form April to August 2005, in twelve rural health facilities (one district hospital and eleven health centres) from Kribi, Mengong, Nkoemvone and Ngoazip, located in the southern province of Cameroon. In this part of Cameroon, located in the rainy equatorial forest, the transmission of malaria is perennial and *Plasmodium falciparum *infection accounts for 95% of malaria cases.

### Study population

Fifty two health professionals (n = 52) were selected including eight medical doctors (15.4%), thirty four nurses (65.4%), and ten health assistants (19.2%). Of these health professionals, 57.7% belonged to public health facilities, 26.9% worked in confessional health centres and 15.4% in private clinics.

### Data collection

Lists of anti-malarial drugs available in public, private health facilities and drugstores were established in each site. Knowledge, attitudes and practices of medical practitioners were collected using a structured questionnaire, previously designed and implemented in two health centres. The questionnaire was divided into four sections including i) evaluation of knowledge and attitudes of medical practitioners regarding treatment guidelines; ii) current use and regimen of anti-malarial drugs as first-line and second-line therapies; iii) knowledge and use of artemisinin derivatives and ACT and iv) sources of information on malaria and anti-malarial drugs.

### Data analysis

Responses from interviews were numerically coded and analysed using Epi-Info version 6.04. Drug costs are expressed in US$. Differences in proportion were analysed using chi-square test when appropriate and significance was set at p < 0.05. This protocol was approved by the operational research board of the Ministry of Public Health, in Cameroon.

## Results

### Availability of anti-malarial drugs and treatment cost

According to the local drug management office, fourteen anti-malarial drugs were available, all of which being generics (Table 1). Three of these drugs, i.e. amodiaquine, quinine and sulphadoxine-pyrimethamine, were used in rural health facilities. Most anti-malarial drugs available in private health structures were trade-named although an agreement has been signed between the national drug office (CENAME) and the private sector. Health professionals usually mentioned the following four trade-named anti-malarial drugs: artemether/lumefantrine (Coartem^®^), dihydroartemisinin (Cotecxin^®^), artesunate (Plasmotrim^®^) and amodiaquine (Camoquin^®^). Chloroquine remained available mainly in confessional health facilities. Public health centres provided the cheapest drugs (p < 0.05). The only AS/AQ available was the blister combination trade-named Arsucam^®^.

**Table 1:** Available anti-malarial drugs and treatment costs in public, private, confessional health facilities and pharmacies

Available anti-malarial drugs	***Mean treatment costs (US $) in health facilities (HF) and private pharmacies***			
	
	Public HF	Private HF	Confessional HF	Pharmacies
AQ oral solution	1.45	3.45	3.27	3.63
AQ 200 mg/tablet	0.22	1.09	0.81	NA
Arsucam^® ^(AS/AQ)	NA	7.63	NA	7.36
Arsumax^® ^(AS)	NA	5.45	2.20	5.54
Artesiane^® ^oral solution	NA	5.81	NA	6.18
CQ 250 mg/tablet	NA	NA	0.72	NA
Cotecxin^® ^oral solution	NA	5.81	5.81	6.78
Fansidar^® ^(SP 525 mg)	0.18	0.90	0.54	1.63
Halfan^® ^oral solution	NA	6.72	6.72	6.72
Halfan^® ^250 mg/tablet	NA	6.36	6.18	6.32
Plasmotrim^® ^50 mg/rectal form	NA	4.36	NA	4.45
Quinine 100 mg/100 ml	2.20	NA	NA	NA
Quinine 100 mg/tablet	0.90	1.81	1.45	NA
Quinine 200 mg/tablet	1.09	NA	1.63	NA
Quinine 300 mg/tablet	1.27	2.00	2.00	1.45
Injectable quinine 400 mg	3.00	5.45	4.36	NA
Injectable quinine 600 mg	2.63	5.45	6.54	NA
Injectable Quinimax^® ^500 mg	4.36	5.45	NA	4.54

NB: all drugs available were not prescribed and therefore, some in the tables are not mentioned in the results.

AQ: amodiaquine; AS: artesunate; CQ: chloroquine; NA: not available.

Artesiane^®^: artesunate; Cotecxin^®^: DiHydroartemisinin; Fansidar^®^: sulphadoxine/pyrimethamine (SP); Halfan^®^: halofantrine; Plasmotrim^®^: artesunate suppositories; Quinimax^®^: quinine.

### New protocol guidelines knowledge by medical practitioners

Four (7.7%) of the fifty two interviewed health professionals knew that AS/AQ was the recommended drug, mentioned in the new guidelines for the treatment of uncomplicated malaria in Cameroon (one was able to provide the official document). According to 92.3% of the medical practitioners, amodiaquine remained the recommended drug. Such recommendation was based upon a national consensus meeting held in February 2004 proposing the use of amodiaquine alone up to availability of AS/AQ by CENAME.

### Adherence to treatment guidelines

After being informed about the new treatment guidelines and about the drugs recommended by the national protocol, thirty-nine health professionals (75.0%) were in favour of the new treatment guidelines and were disposed to prescribe AS/AQ combination if available. Among the remaining practitioners (n = 13), eleven (21.0%) declined to accept treatment guidelines based on the presumed adverse events attributed to amodiaquine when used in monotherapy (mainly asthenia or/and vomiting). Quinine or artesunate alone was prescribed by 4.0% of the practitionners.

### Attitudes related to AS/AQ combination therapy

With regards to AS/AQ combination therapy, nineteen of the fifty two health professionals (36.5%) complained about the lack of paediatric forms (suppositories and oral solutions) which was considered as a crucial problem for children's prescription. 34.6% thought that, drugs with an estimated price of 7.5 US$ were too expensive for poor rural patients. 26.9% of the practitioners suggested a reduction in the number of artesunate and amodiaquine tablets per dose while 25.0% argued for a reduction of the dosage of amodiaquine alone. 19.2% mentioned a lack of information related to the use of ACT in the protocol guidelines; 5.8% indicated that the choice of white colour (for artesunate tablets) and yellow colour (for amodiaquine tablets) was not wise, as it was difficult to differentiate between the two under low light. 5.8% complained about the absence of pre-packaged drugs for home management of malaria and 2.0% proposed a systematic administration of antibiotics with malaria treatment.

### Anti-malarial drugs used as first-line treatment

Quinine was the most prescribed anti-malarial drug for adult patients. Tablets (300 mg) and parenteral quinine were respectively used by 65.3% and 7.6% of the prescribers while 71.2% preferred tablets for pregnant women (Table 2). 2.0% of the physicians administered the recommended combination therapy (AS/AQ) to adults. No artemisinin-based combination therapy has been mentioned for pregnant women.

**Table 2:** Drugs used as first-line treatment by health professionals in rural zone

Anti-malaria drugs	Adults (%)	Pregnant women (%)	Children under 5 (%)
Arsucam^® ^(AS/AQ)	**2.0**	0.0	**2.0**
Quinine tablets	**65.3**	**71.2**	**23.0**
Amodiaquine	21.1	15.4	**67.3**
Chloroquine	2.0	0.0	0.0
Injectable quinine	7.6	7.6	5.7
Sulphadoxine/Pyrimethamine	2.0	3.8	0.0
Amodiaquine+SP	0.0	2.0	0.0
Artemether (injectable)	0.0	0.0	2.0

Concerning children with suspected malaria, nurses administered oral solutions of amodiaquine (67.3%) or quinine (23.0%). Injectable quinine was prescribed as first-line drug by 5.7% of nurses and 2.0% declared having prescribed the recommended AS/AQ.

### Anti-malarial drugs used as second-line treatment

According to thirty-two prescribers (61.5%), persistent fever occurring at day 3 post-treatment (associated or not with other clinical signs) was the main criteria to evaluate therapeutic failure (Table 3). In that case, 36.4% performed a thick blood smear test prior to treatment using another anti-malarial drug. 53.3% directly used injectable quinine in adults (68.8%), pregnant women (84.4%) or children (48.0%). Moreover, 2% prescribed antibiotics. Although nurses tended to use more injectable drugs than doctors, the differences in prescribing patterns were not significant (p = 0.08).

**Table 3:** Drugs used as second-line treatment by health professionals in rural zone

Anti-malarial drugs	Adults (%)	Pregnant women (%)	Under-five children (%)
Injectable artemether	**2.0**	0.0	**2.0**
Injectable quinine	**68.8**	**84.4**	**48.0**
Amodiaquine	2.0	0.0	3.9
Artesunate	3.9	2.0	0.0
Dihydroartemisinin	2.0	2.0	7.6
Halofantrine	2.0	0.0	2.0
Quinine tablets	9.6	7.6	32.5
Injectable quinine+SP	2.0	0.0	0.0
Quinine + antibiotics	2.0	2.0	2.0
Sulphadoxine/pyrimethamine	5.7	2.0	2.0

SP: sulphadoxine/pyrimethamine

Health professionals mentioned that severe anaemia (78.6%) and/or signs of cerebral malaria (42.9%) were the main observed complications usually referred to specialized hospitals.

### Use of artemisinin derivatives and ACT

Twenty-one (40.4%) of the fifty two health professionals never used artemisinin derivatives since they started working in rural health facilities (Table 4). Artemisinin derivatives, including artesunate, dihydroartemisinin and artemether, were prescribed as monotherapy (44.3%) or as both monotherapy and bitherapy (15.3%). Artesunate and dihydroartemisinin alone were found as the most prescribed drugs. Artemether/lumefantrine was the first artemisinin-based combination mentioned by prescribers in rural district hospitals. However, this drug was not available in drugstores and pharmacies at the time of the study. About 62% of the prescribers did not use artemisinin derivatives for various reasons: (1) non-availability of the drugs in rural areas; (2) only partial information (19%); (3) high cost (14.3%); (5) treatment efficacy being suspected to be lower than the currently used quinine (4.8%).

**Table 4:** Artemisinin derivatives prescribed (cited) by health professionals in rural zones

Artemisinin derivatives	(Number) % of prescribers
**Not used (n = 21)**	(21) 40.4
**Used (n = 31)**	
***Monotherapies***	
Artesunate	(11) 21.2
Dihydroartemisinin	(9) 17.3
Artemether	(3) 5.8
***Bitherapies (ACT)***	
Coartem^®^	(6) 11.5
Arsucam^®^	(2) 3.8

On the other hand, 7.7% of health professionals declared prescribing drugs according to their trade names and 5.8% were influenced by patient's requests (Table 5).

**Table 5:** Main selection criteria of artemisinin derivatives according to prescribers (n = 52)

Choosing criteria	(Number) % of prescribers
Therapeutic efficacy	(18) 34.6
Observance (few tablets)	(14) 26.9
Few adverse effects	(7) 13.5
Drug cost (lowest price)	(6) 11.5
Name of drugs	(4) 7.7
Patient's request	(3) 5.8

### Main sources of malaria drugs information in rural health facilities

A total of eight physicians and two nurses (19.2% of the personnel) had followed training sessions on malaria treatments. In rural health facilities, prescribers usually obtained information about malaria treatment and drugs from their colleagues (40.4%) and from medical visitors employed by private pharmaceutical providers (36.5%). Only 5.8% had participated to a training seminar organized by the Ministry of Public Health of Cameroon within the last two years (Table 6).

**Table 6:** Main sources of information in rural health facilities

Sources of information	(Number) % of prescribers
Health personnel	(21) 40.4
Medical visitors	(20) 36.5
Medias	(5) 9.6
Medical reviews	(4) 7.7
Ministry of public health	(3) 5.8

## Discussion

Reporting baseline data at the time of introduction of ACT provides useful information to highlight the strength and weaknesses of implementation programmes, and set up proper key progress indicators. The present survey highlights some of the difficulties related to the implementation of new national guidelines for the treatment of malaria in rural areas, where drug availability is restricted to public health facilities, religious health institutions, and a few drugstores. Our findings are in agreement with a previous study conducted in Zambia [3], indicating that isolated rural areas, the most concerned by malaria prevalence, have difficulties to be educated about treatment progresses and have limited access to official information. Moreover, negative attitudes from doctors and medical assistants toward the national guidelines were reported, mainly based upon mild adverse events attributed to amodiaquine (asthenia and vomiting), and rare deadly adverse events (agranulocytosis) when previously used as a prophylactic drug, leading to its market withdrawal for many years. Such observations should be taken into consideration, as the new 3-days regimen of AS/AQ was implemented in 2007 in Cameroon, made of a single daily bicoloured tablet; yellow (artesunate) and white (amodiaquine).

The lack of impact of the national malaria treatment guidelines on the behaviour of medical practitioner has previously been reported in Burkina Faso [4] and Sudan [5]. Quinine has already been referred as the most prescribed first-line anti-malarial drug (84.4%) in public health institutions in Cameroon [6]. These findings suggested that ensuring the availability of AS/AQ in the public sector will not be the only incentive to ensure proper use and compliance to this regimen. Nationwide information on the rational use of anti-malarial drugs must be provided together with proper training of health professionals in order to obtain their full participation and understanding. In addition, the current widespread availability of various artemisinin monotherapies from the private sector and the presence of private medical representatives from pharmaceutical companies might influence specific prescriptions in each health facility. Therefore, training, information and early implication of health professionals in decision analysis should strongly improve adherence to newly proposed guidelines for the treatment of malaria.

At the time of the study, in August 2005, the use of AS/AQ was not implemented in rural areas of southern Cameroon despite the official publication and access to the new guidelines in February 2004. However, for the treatment of uncomplicated malaria, a majority of health professionals will favour the present combination if appropriately formulated (paediatric forms and reduced pills intake) and offered at affordable price.

Modifying treatment protocols is a complex issue. The greatest challenge is an early supply of new drugs, at low price, provided in all health institutions preceded by the proper information in order to convince health professionals to adhere to new clinical practices. In addition, following a proposal from WHO, a public release of the Cameroonian Ministry of Public Health announced that all anti-malarial drugs available in monotherapy should not be sold and used anymore in health facilities. Therefore, nationwide withdrawal of these drugs was performed in public and private pharmacies and drugstores, starting January 2007. Such a measure might prove efficient if (i) the recommended AS/AQ formulation will be made available at the national level with adapted paediatric formulations and (ii) reasons why monotherapies are not recommended anymore are clearly explained to the overall population.

Furthermore, in February 2007, the cost of malaria treatment was reduced to a price between 0.30 US$ and 1.3 US$ in public hospitals and health centres, therefore, improving access to treatment for the rural population.

## Conclusion

Prescribers should be involved as soon as possible in projects related to the optimization of treatment guidelines and comply with new drugs. This baseline information could be used as the starting points for further monitoring and evaluation of artemisinin-based combination therapy in Cameroon.

## Competing interests

The authors declare that they have no competing interests.

## Authors' contributions

CS, MG, NVL, DM, DB and PM designed the study. CS, MG, PM performed the field work. CS, MG, NVL and PM analysed and interpreted the results. CS and PM wrote the paper. CS, CS, DM and PM reviewed the article. All authors read and approved the final manuscript.

## Disclaimer

The views expressed in this paper are solely those of the authors. Trade names are used for identification only and do not represent endorsement by the National Malaria Control Programme. There are no conflicts of interest in regards to this work.
